# Single-shot diffraction data from the Mimivirus particle using an X-ray free-electron laser

**DOI:** 10.1038/sdata.2016.60

**Published:** 2016-08-01

**Authors:** Tomas Ekeberg, Martin Svenda, M. Marvin Seibert, Chantal Abergel, Filipe R.N.C. Maia, Virginie Seltzer, Daniel P. DePonte, Andrew Aquila, Jakob Andreasson, Bianca Iwan, Olof Jönsson, Daniel Westphal, Duško Odić, Inger Andersson, Anton Barty, Meng Liang, Andrew V. Martin, Lars Gumprecht, Holger Fleckenstein, Saša Bajt, Miriam Barthelmess, Nicola Coppola, Jean-Michel Claverie, N. Duane Loh, Christoph Bostedt, John D. Bozek, Jacek Krzywinski, Marc Messerschmidt, Michael J. Bogan, Christina Y. Hampton, Raymond G. Sierra, Matthias Frank, Robert L. Shoeman, Lukas Lomb, Lutz Foucar, Sascha W. Epp, Daniel Rolles, Artem Rudenko, Robert Hartmann, Andreas Hartmann, Nils Kimmel, Peter Holl, Georg Weidenspointner, Benedikt Rudek, Benjamin Erk, Stephan Kassemeyer, Ilme Schlichting, Lothar Strüder, Joachim Ullrich, Carlo Schmidt, Faton Krasniqi, Günter Hauser, Christian Reich, Heike Soltau, Sebastian Schorb, Helmut Hirsemann, Cornelia Wunderer, Heinz Graafsma, Henry Chapman, Janos Hajdu

**Affiliations:** 1 Department of Cell and Molecular Biology, Laboratory of Molecular Biophysics, Uppsala University, Husargatan 3 (Box 596), SE-751 24 Uppsala, Sweden; 2 Center for Free-Electron Laser Science, DESY, Notkestrasse 85, 22607 Hamburg, Germany; 3 Information Génomique et Structurale (UMR7256) CNRS & Aix-Marseille Université, Institut de Microbiologie de la Méditerranée (FR3479), Parc Scientifique de Luminy, Case 934, 13288 Marseille Cedex 9, France; 4 LCLS, SLAC National Accelerator Laboratory, 2575 Sand Hill Road, Menlo Park, California 94025, USA; 5 European XFEL, Albert-Einstein-Ring 19, 22761 Hamburg, Germany; 6 Commissariat à l’énergie atomique et aux énergies alternatives, Centre d’études de Saclay, 91191 Gif sur Yvette cedex, France; 7 Molekyl- och kondenserade materiens fysik, Institutionen för Fysik och Astronomi, Uppsala University, Lägerhyddsvägen 1 (Box 524), SE-751 20 Uppsala, Sweden; 8 The University of Melbourne, 161 Barry Street, Parkville, 3010 Victoria, Australia; 9 Photon Science, DESY, Notkestrasse 85, 22607 Hamburg, Germany; 10 Stanford PULSE Institute, SLAC National Accelerator Laboratory, 2575 Sand Hill Road, Menlo Park, California 94025, USA; 11 Centre for BioImaging Sciences, National University of Singapore, 14 Science Drive 4 Blk S1 A, Singapore 117546, Singapore; 12 Synchrotron SOLEIL, L’orme des Merisiers roundabout of St Aubin, 91190 Saint Aubin, France; 13 Lawrence Livermore National Laboratory, 7000 East Avenue, Mail Stop L-211, Livermore, California 94551, USA; 14 Max-Planck-Institut für medizinische Forschung, Jahnstr. 29, 69120 Heidelberg, Germany; 15 Max Planck Advanced Study Group, Center for Free Electron Laser Science, Notkestrasse 85, 22607 Hamburg, Germany; 16 Max-Planck-Institut für Kernphysik, Saupfercheckweg 1, 69117 Heidelberg, Germany; 17 Department of Physics, J.R. Macdonald Laboratory, Kansas State University, 116 Cardwell Hall, Manhattan, Kansas 66506, USA; 18 PNSensor GmbH, Otto-Hahn-Ring 6, 81739 Munich, Germany; 19 Max-Planck-Institut Halbleiterlabor, Otto-Hahn-Ring 6, 81739 Munich, Germany; 20 Max-Planck-Institut für extraterrestrische Physik, Giessenbachstrasse, 85741 Garching, Germany; 21 Universität Siegen, Emmy-Noether Campus, Walter Flex Str. 3, 57068 Siegen, Germany; 22 Physikalisch-Technische Bundesanstalt, Bundesallee 100, 38116 Braunschweig, Germany; 23 Institut für Optik und Atomare Physik, Technische Universität Berlin, Hardenbergstrasse 36, 10623 Berlin, Germany; 24 University of Hamburg, Notkestrasse 85, 22607 Hamburg, Germany

**Keywords:** Biological physics, Virus structures, X-rays

## Abstract

Free-electron lasers (FEL) hold the potential to revolutionize structural biology by producing X-ray pules short enough to outrun radiation damage, thus allowing imaging of biological samples without the limitation from radiation damage. Thus, a major part of the scientific case for the first FELs was three-dimensional (3D) reconstruction of non-crystalline biological objects. In a recent publication we demonstrated the first 3D reconstruction of a biological object from an X-ray FEL using this technique. The sample was the giant Mimivirus, which is one of the largest known viruses with a diameter of 450 nm. Here we present the dataset used for this successful reconstruction. Data-analysis methods for single-particle imaging at FELs are undergoing heavy development but data collection relies on very limited time available through a highly competitive proposal process. This dataset provides experimental data to the entire community and could boost algorithm development and provide a benchmark dataset for new algorithms.

## Background & Summary

Free-electron lasers (FEL) provide ultra short and extremely bright pulses of coherent X-rays^1^. It has been predicted that such pulses could enable structure determination without crystallization by outrunning radiation damage and thus capturing diffraction data before the particle has time to respond and eventually be destroyed by the deposited energy^2^. Experimental verification of this ‘diffraction-before-destruction’ principle has been demonstrated several times for resolution down to 10 nm (refs 3,4).

Using many such diffraction patterns from multiple copies of a reproducible sample, the patterns could be assembled into a 3D diffraction space from which the 3D structure could be derived^5,6^. This promise was a main part of the scientific case for building free-electron lasers^7^. Several examples of 2D reconstructions from biological samples at X-ray FEL have been demonstrated^4,8,9^ but 3D reconstructions have remained elusive.

A single diffraction pattern represents a curved slice through the Fourier transform of the electron density of the object. For successful 3D reconstruction many diffraction patterns from identical particles need to be assembled into the complete 3D Fourier transform of the particle. This is difficult since the orientation of the injected particles is unknown and has to be recovered from the diffraction data alone. A recent paper^10^ demonstrates this, using a modified version of the expand, maximize and compress algorithm^5^ (EMC) on the Mimivirus particle. Here we describe the data collection, data preprocessing and the dataset that was used for this reconstruction.

Today, beam time at free-electron lasers is scarce as there are few facilities and they serve a multitude of scientific disciplines. Furthermore, applications such as 3D imaging require a large amount of effort in algorithm development and testing. Several groups around the world are active in this development but the majority of them don’t have regular beam time access. This dataset can thus serve as a benchmark for algorithm testing and give many more groups access to experimental data.

For any new method, validation tools are of crucial importance. Therefore, together with the 3D reconstruction of the Mimivirus we also presented two new validation methods^10^. Further development of these methods, and the development of new ones will therefore benefit from being applied to this dataset in particular.

The sample in this dataset is the Mimivirus (*Acanthamoeba polyphaga mimivirus*)^11,12^. Mimivirus is part of a recently discovered class of giant DNA viruses. Viral capsid is pseudo-icosahedral with a corner-to-corner diameter of 500 nm and a face-to-face diameter of 400 nm (ref. 13). The virus is covered by fibres with a length of 125 nm giving it a total diameter of 750 nm (ref. 14).

## Methods

These methods were described in ref. 10. The description here is more detailed with regards to data collection and on-line data analysis.

### Sample injection

Purified Mimivirus particles^15^ were transferred into a volatile buffer (250 mM ammonium acetate, pH 7.5) and the suspension was aerosolized with helium in a gas dynamic nebulizer^16^. The aerosol of hydrated and adiabatically cooled particles entered a differentially pumped aerodynamic lens^17^.

### Data collection

Experiments were performed at the Atomic Molecular Optics (AMO) beam line^18^ of the Linac Coherent Light Source (LCLS) hard X-ray laser^1^, using the CAMP^19^ instrument^20,21^. The experiment was part of an experiment running from June 17 to June 21 of 2010 with proposal number L150. Diffraction data were recorded on a pair of pnCCD detectors^19^ at a repetition rate of 60 Hz matching the repetition rate of LCLS. The two detectors were placed at a distance of 740 mm from the interaction region with a gap between them of 2.1 mm to let the direct beam through. The pixel size is 75 μm and each detector in the pair has 512×1,024 pixels giving the full setup a pixel count of 1,024×1,024 pixels.

The photon energy was 1.2 keV corresponding to a wavelength of 1.03 nm. At this wavelength the full-period resolution at the edge of the detector is 19.9 nm. The electron bunch used to create the X-ray pulse was 70 fs long (full duration at half maximum) and the X-ray pulse is believed to be shorter than this^22^. The focus was ~10 μm (full width at half maximum) at the interaction point, giving a power density of ~3.4 10^15^ W cm^−2^ or 10^12^ photons per pulse.

The experiment was performed at a pressure of 10^−6^ mbar to reduce background scattering. Some of the most important experiment parameters are summarized in Table 1.

### On-line data analysis

On-line hit-identification provided real-time statistics that guided injector alignment and tuning. Hits with a high scattering strength were identified by counting the number of pixels that measured a value above a threshold. Diffraction patterns with more than 500 pixels with a value above 170 ADU were defined as a hit. See ref. 21 for a detailed description.

## Data Records

Two datasets are provided: the full record of all collected data and a smaller preprocessed dataset. Both data sets are available in the same CXIDB entry (Data Citation 1).

### Full data record

We provide all data collected from the Mimivirus at this beamtime before any preprocessing or sorting. This dataset is in the extended tagged container (XTC) file format. This can be converted to HDF5 format using programs such as CASS^23^ or Cheetah^24^. This conversion normally also involves preprocessing and the data is therefore provided in the untouched XTC format.

The record contains 19 LCLS runs. 14 of these had the sample injector and X-ray laser turned on while the remaining 5 runs only collected detector background noise. These so called ‘dark runs’ can be used for to better subtract the background from the actual data. Table 2 shows a list of all 19 runs.

Initial hit finding showed that 0.3% of the frames contained diffraction that was stronger than the background. The rest were misses, i.e., frames that were read out when the pulse did not hit any particle. In addition to hits from single Mimivirus particles the hits also include droplets of buffer, clusters of viruses and a few particles that were injected earlier and had stayed in the injection system.

### Preprocessed and filtered data

This dataset contains the 198 preprocessed (see ref. 19) diffraction patterns that were used in ref. 10 to recover the 3D structure of the Mimivirus particle. This data is in the CXIDB format described in ref. 25. The pixel values in the data are in arbitrary detector units (ADU). The conversion factor from ADU to number of photons is 7 ADU per photon. Some areas of the detector were unreadable and some scattering angles were not covered by the detector, such as the gap between the two detector halves that lets the direct beam through. These areas are identified by a *mask* entry in the CXIDB file format.

**Figure 1:**
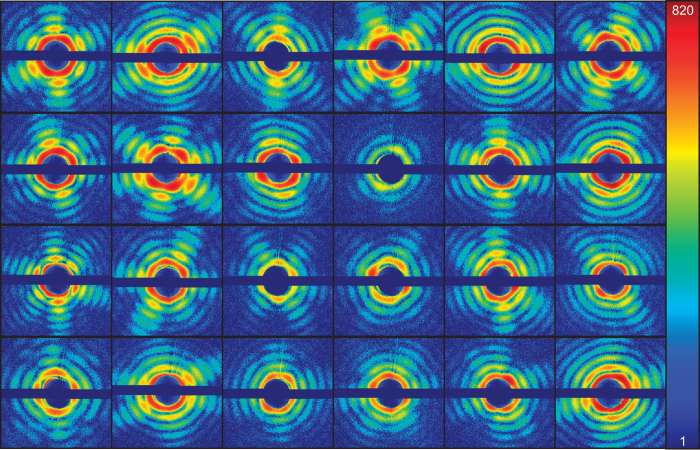
The first 24 of the 198 diffraction patterns in this dataset. The color scale is logarithmic and ranges from 1 to 820 photons per pixel. This is a modification of a figure previously presented in ref. 10.

The data was filtered in three steps. (1) Hits were distinguished from blanks using methods described in refs 21 and 24. This yielded 1,600 hits. (2) 307 Diffraction patterns that correspond to a Mimivirus particle were selected by hand. An icosahedral or pseudo-icosahedral particle will in most orientations yield a distinctive type of pattern showing six outwards-going streaks. This feature and particle size, determined from the fringe spacing, was used for this selection. (3) When the detector is exposed to high intensities, the intensity can spill over from a pixel to neighboring pixels. We start seeing these effects at intensities above 750 photons per pixel. In the final dataset, diffraction patterns suffering from this effect were filtered out, resulting in 198 unsaturated diffraction patterns. A subset of this dataset is shown in Fig. 1.

## Technical Validation

### 3D reconstruction

The 198 diffraction patterns were successfully assembled in a 3D Fourier volume and subsequent phase retrieval provided the full 3D electron density of the virus with a full-period resolution of 125 nm^10^. This indicates that the Mimivirus is reproducible to at least this resolution.

### Validation of the 3D reconstruction

In cryo-electron microscopy (cryo-EM), data is routinely split prior to analysis and the analysis is performed in parallel on the two sets^26^. Our first method for validating the 3D reconstruction is an adaptation of this technique. The diffraction patterns are randomly split in two sets of equal size. The recovery of the 3D alignment is performed independently on the two sets using the same parameters but independent random starting points. Phase retrieval is also performed independently using the same parameters. The standard practice in the field is to repeat the reconstruction at least 100 times and the results are then averaged to average out effects of the random starting point^4,27^.

The EMC algorithm recovers the relative orientation of the particles from the diffracted data alone but the arbitrary rotation of the entire system can be different comparing the two resulting 3D electron density maps. In order to compare the two recovered electron densities we therefore have to rotate one of the two data sets to best match the other. This is done using brute force by interpolating one of the maps at a regular array corresponding to the tested rotation. To compare the two maps, the Pearson correlation coefficient is calculated. The rotation with the highest Pearson correlation is assumed to correspond to the proper relative orientation.

The two aligned electron density maps are then compared using the Fourier shell correlation (FSC)^26^, which provides a measure of the similarity as a function of resolution. The threshold for what is regarded as an acceptable fit ranges between 0.14 and 0.5 in cryo-EM literature^28,29^.

In X-ray crystallography some Bragg spots are usually excluded from the analysis and instead that information is used to verify the recovered expected strength of the respective Bragg spot^30^. Using the same idea, 10% of the diffraction patterns were selected to be used for validation only. In the EMC scheme these patterns are excluded from the analysis but are still compared to the recovered Fourier transform of the particle. The measure for determining whether the recovered model agrees with the excluded diffraction patterns is the likelihood function used internally in EMC.

The analogy with the R_free_ value in crystallography should not be over emphasized. Bragg peaks are linearly independent parameters and ther is no suitable analogy in the continuous diffraction case. Furthermore, in the case described here, the process that is validated is that of pattern alignment and not phase retrieval. It is therefore natural to choose individual diffraction patterns as the information unit to exclude rather than i.e., individual pixels or regions of pixels.

These validation methods were previously described in ref. 10.

## Usage Notes

Data was stored in the CXIDB^25^ data format which uses the HDF5 format. HDF5 files are readable in many computing environments including python using the h5py module and MATLAB using e.g., the h5read function. Convenient functions for accessing the CXIDB data file exist in the libspimage package for C and python^31^. For visualizing data the CXIDB file browser Owl (https://github.com/FilipeMaia/owl) is recommended.

## Additional Information

**How to cite this article:** Ekeberg, T *et al.* Single-shot diffraction data from the Mimivirus particle using an X-ray free-electron laser. *Sci. Data* 3:160060 doi: 10.1038/sdata.2016.60 (2016).

## Supplementary Material



## Figures and Tables

**Table 1:** Summary of experimental parameters.

**Parameter**	**Value**
Photon energy	1,200 keV
Detector distance	0.74 m
Pixel size	75 μm
Number of pixels	1,024×1,024
Focal size	10 μm^2^

**Table 2:** List of experimental runs.

**Run number**	**Sample**	**Number of frames**	**Number of frames selected for analysis in ref. 10**
73	Dark	651	N/A
80	Mimivirus	14,273	0
81	Mimivirus	14,450	0
82	Mimivirus	11,371	0
83	Dark	15,636	N/A
84	Mimivirus	65,594	0
87	Mimivirus	93,840	0
89	Dark	3,821	N/A
90	Mimivirus	77,644	30
91	Mimivirus	4,943	6
92	Mimivirus	33,721	39
93	Mimivirus	43,679	24
94	Mimivirus	58,931	40
95	Mimivirus	42,083	10
97	Mimivirus	36,899	33
98	Dark	7,794	N/A
152	Dark	2,498	N/A
156	Mimivirus	64,977	3
157	Mimivirus	90,403	13
Runs labeled as dark had the X-ray beam turned off and are included to allow for detector calibration. The lack of good hits before run 90 was possibly fixed by a changed injection nozzle at this point.			
